# When Parotid Swelling Reveals Relapsed Breast Cancer: A Rare Metastatic Pathway

**DOI:** 10.1002/ccr3.71393

**Published:** 2025-11-21

**Authors:** Ferchichi Sana, Kharrat Ghada, Siwar Sbaihi, Halwani Chiraz

**Affiliations:** ^1^ ENT Department, Faculty of Medicine of Tunis, Mohamed Taher Maâmouri University Hospital University of Tunis El Manar Tunis Tunisia; ^2^ Radiology Department, Faculty of Medicine of Tunis, Mohamed Taher Maâmouri University Hospital University of Tunis El Manar Tunis Tunisia; ^3^ ENT Department, Faculty of Medicine of Tunis, Military Hospital of Instruction of Tunis University of Tunis El Manar Tunis Tunisia

**Keywords:** ear nose and throat, general medicine, oncology, pathology and laboratory medicine, surgery

## Abstract

Parotid tumor can be the first manifestation of relapsed breast cancer, even years after remission. Awareness of this rare metastatic pathway is crucial, as timely recognition of atypical parotid masses in patients with a breast cancer history can guide prompt diagnosis and improve management outcomes.

## 1 Introduction

The majority of secondary parotid gland tumors represent regional metastases originating from cutaneous squamous cell carcinoma or malignant melanoma of the head and neck region. In addition, regional parotid metastases may occasionally arise from primary tumors of the upper aerodigestive tract, conjunctiva, lacrimal gland, or thyroid gland [1].

Approximately 20% of secondary parotid tumors correspond to distant metastases or to metastases from carcinomas or melanomas of unknown primary origin. Nevertheless, distant metastatic involvement of the parotid gland remains exceptionally rare [1].

Among women, breast cancer is the most prevalent malignancy worldwide. Although it commonly metastasizes to the bones, liver, and lungs, dissemination to unusual sites may occur and often poses significant diagnostic and therapeutic challenges [2]. In this context, parotid gland metastasis from breast carcinoma is exceedingly uncommon, and its occurrence as a presentation or indicator of disease recurrence is particularly rare.

However, identifying this unusual presentation is essential, and it may serve as an important clue in diagnosing breast cancer relapse.

Herein, we present a rare case in which an isolated parotid tumor of an otherwise healthy woman was the first indicator of relapsing breast cancer.

## 2 Case History/Examination

**FIGURE 1:**
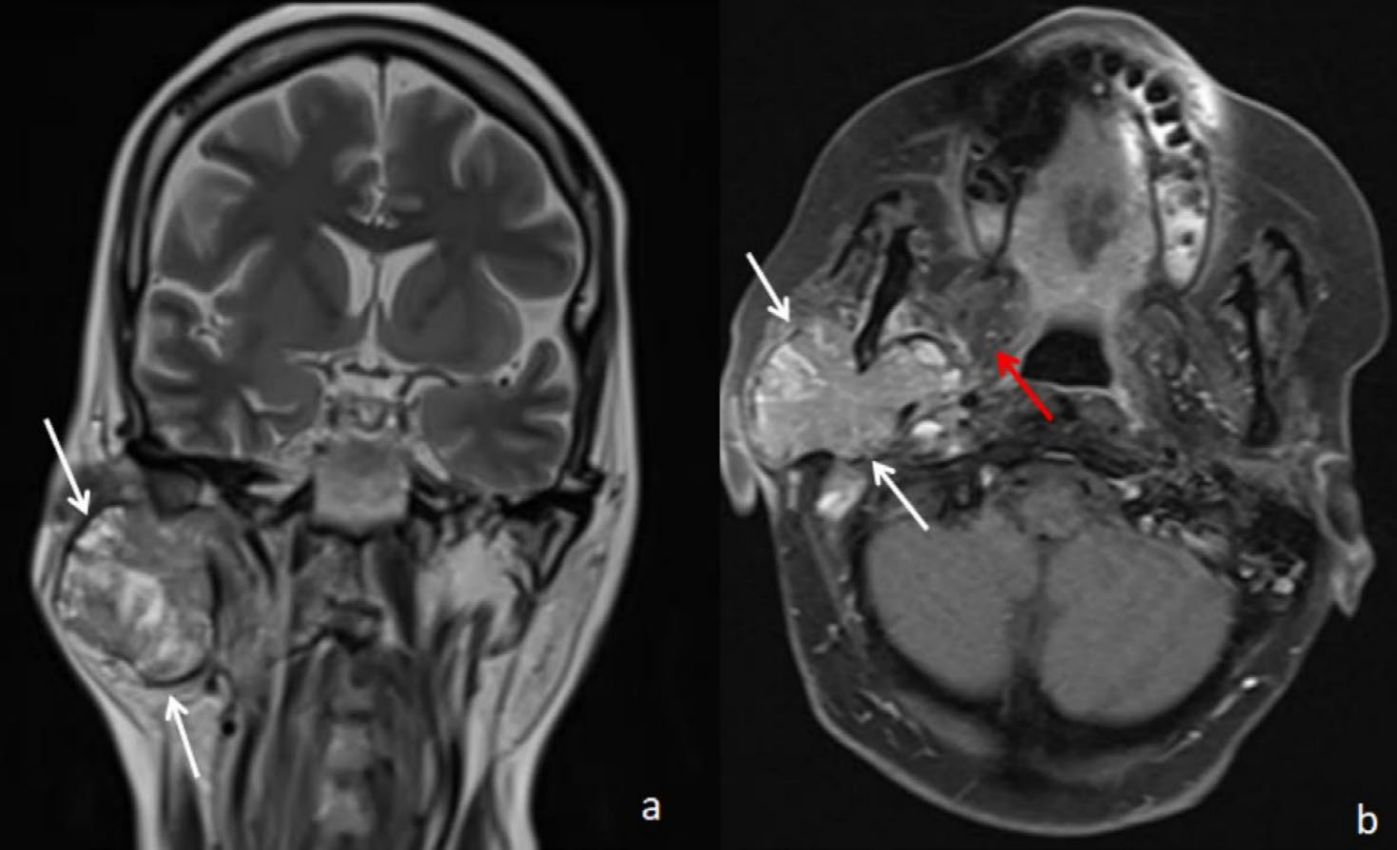
Coronal T2‐weighted sequence (a) and T1 with saturation of the fat signal after injection of Gadolinium in axial section (b), showing a large mass occupying the whole of the right parotid gland (white arrows), with intermediate and heterogeneous signal in T2 and intensely and heterogeneously enhanced after injection of Gadolinium. The mass extends into the deep lobe of the parotid gland and displaces the medial pterygoid muscle medially and anteriorly (red arrow).

A 52‐year‐old woman with a history of breast cancer, treated 5 years earlier by Patey mastectomy followed by adjuvant chemoradiotherapy, had favorable evolution. She presented to the ENT department of Mohamed Taher Maâmouri University Hospital. Her main complaint was a rapidly growing right preauricular swelling that had been evolving for 2 months. Clinical examination revealed a painless, firm, five‐centimeter mass in the right parotid region with no inflammatory signs. There was no facial nerve palsy. The cervical lymph node examination was unremarkable. MRI revealed a tissue mass affecting the right parotid gland's superficial and deep lobes, with an enlarged stylomandibular foramen and encapsulated involvement of the styloid process. The mass displayed irregular margins, with blurred areas and a heterogeneous signal on T1 and T2 sequences, showing signs of necrosis and hemorrhage (Figure 1). Additionally, there was a lesion on the right mandibular condyle and lesions on the C1 and C2 vertebrae with a secondary appearance. The ADC value was 0.74, and the enhancement curve followed a type C pattern. A twice‐performed Fine Needle Aspiration (FNA) was inconclusive due to a paucicellular and nonrepresentative sample. In addition, the core needle was not available. Given the high suspicion of malignancy, a biopsy was performed under local anesthesia. The histopathological analysis identified characteristics consistent with breast carcinoma metastasis (invasive ductal carcinoma). Immunohistochemical staining showed a hormone receptor‐negative profile. A full‐body scan showed no evidence of local breast cancer recurrence or additional metastatic lesions.

Given the exceedingly rare occurrence of parotid metastasis from breast cancer, no standardized treatment guidelines exist. In our patient, the presence of multiple lesions involving the cervical vertebrae, the condyle, and a diffusely infiltrated, unresectable parotid gland prompted the tumor board to pursue combined chemoradiotherapy to achieve local control and address systemic disease. The clinical course was complicated by hematologic and infectious toxicities, culminating in severe neutropenia, septicemia, and death 4 months after treatment.

## 3 Discussion

The head and neck region, because of its abundant vascularization and extensive lymphatic network, may occasionally serve as an unusual site for metastatic spread from a distant primary tumor. In this context, we may cite the example of uncommon parapharyngeal metastases, which have been reported to originate from squamous cell carcinoma at a distant primary site [3].

The parotid gland is an uncommon site for metastatic disease, and when metastases do occur, they most often originate from head and neck primaries, with malignant melanoma and squamous cell carcinoma being the most frequent sources [1]. Metastases from non‐head and neck sites are rare, due to differences in lymph node distribution, anatomical relationships, and lymphatic drainage. Among these rare cases, breast cancer metastasis to the parotid gland is exceptionally uncommon and can present either synchronously or metachronously—even up to 32 years after the initial diagnosis—regardless of the primary disease stage or adequacy of initial treatment [4].

According to the World Health Organization (WHO), breast cancer is the most commonly diagnosed cancer worldwide, with over 2.3 million new cases in 2020. It is also the leading cause of cancer death among women, accounting for approximately 685,000 deaths in 2020 [5]. Breast cancer typically appears as a painless lump in the breast or axilla, often characterized by a firm, immobile, irregular, or fixed texture [6].

More rarely, the first noticeable sign may be a distant metastasis. The bone is the most common metastatic site overall, followed by the lung, liver, and brain [2]. While extremely rare, breast cancer can spread to the parotid gland. The first documented case was reported by Abrams et al. in 1950, based on an autopsy review of 167 breast cancer cases, where only one instance of parotid metastasis was identified [7]. Between 1982 and 2017, only 21 cases were recorded globally [8]. Metastasis to other salivary glands, such as the submandibular gland, is even less common [9].

The majority of authors agree that the metastatic spread of breast cancer to the parotid gland occurs primarily through a hematogenous route. However, lymphatic dissemination via the thoracic duct is also possible [8].

This metastasis usually occurs in the later stages of the disease. The originality of our case report lies in the fact that parotid metastasis was the first clinical sign of breast cancer recurrence, occurring 5 years after the patient was declared in remission.

Metastases from different types of breast cancer, including invasive ductal carcinoma, invasive lobular carcinoma, and even malignant phyllodes tumor, have been reported. Among these, invasive ductal carcinoma is the most frequently observed, as seen in our patient [10].

Clinically, patients often present with a periauricular mass, which may or may not be accompanied by peripheral facial nerve palsy.

MRI is the preferred imaging modality for assessing parotid gland tumors.

MRI outperforms ultrasonography and CT in detecting lesions and visualizing surrounding tissue structures. The comprehensive data provided by MRI, such as lesion shape, signal characteristics, and contrast behavior, significantly improve diagnostic precision [11].

FNA is considered a reliable and precise primary diagnostic tool [12]. It has an 85% accuracy rate in distinguishing malignant from benign lesions and differentiating primary parotid neoplasms from metastatic ones10. However, the twice‐performed FNA in our patient was noncontributory. In fact, FNA may be insufficient for diagnosing parotid metastases of breast cancer, as cellular yield may be low and cytological features can overlap with those of primary salivary gland tumors [13]. Moreover, the absence of preserved tissue architecture limits the ability to perform essential immunohistochemical analyses (ER, PR, HER2, GATA3, and mammaglobin) required to confirm mammary origin. Given the rarity of parotid metastases and the diagnostic challenges of cytology alone, tissue biopsy remains the gold standard when clinical and radiological suspicion is high [13].

In the present case, given the patient's personal history of breast cancer and the high radiological suspicion of malignancy (parotid tumor characteristics, vertebrae and mandibular condyle lesions), we decided to perform a surgical tumor biopsy under local anesthesia, which confirmed breast cancer metastasis (invasive ductal carcinoma).

Given the rarity of this metastatic involvement, no published treatment guidelines have been developed. However, most authors agree that the approach differs depending on whether the parotid metastasis is isolated or associated with other metastases.

Most authors recommend a parotidectomy (total or superficial) with negative margins for a single parotid metastasis, preferably preserving the facial nerve whenever possible. Although some authors advocate for an ipsilateral functional neck dissection, most consider it unnecessary, as the metastatic spread is primarily hematogenous. Most authors also recommend adjuvant postoperative radiotherapy for the parotid region and the neck [14].

In cases of multisite metastases, palliative chemotherapy or concurrent chemoradiotherapy may be considered. Targeted therapy (e.g., Herceptin for HER2‐positive tumors) may be proposed if applicable [15].

Despite the available treatments, patients with metastatic parotid gland involvement generally have a poor prognosis, with a reported 5‐year survival rate of only 10% [12]. However, it should be noted that patients with isolated metachronous parotid metastasis may have a potentially better prognosis, particularly if there is a long interval between breast cancer and metastasis [14].

## 4 Conclusion

Parotid metastasis in breast cancer is an infrequent occurrence, particularly when it serves as the initial sign of a breast cancer relapse. A personal history of breast cancer, even if considered disease‐free, should raise the clinician's suspicion of a potential synchronous or metachronous metastasis.

Recognizing such atypical presentations can significantly impact the progression of the disease and lead to more favorable outcomes.

## Author Contributions

**Ferchichi Sana:** conceptualization, methodology, writing – original draft. **Kharrat Ghada:** investigation. **Siwar Sbaihi:** resources, validation. **Halwani Chiraz:** validation, writing – review and editing.

## Ethics Statement

Our institution does not require ethical approval for reporting individual cases or case series. We anonymously reported clinical and imaging information concerning our patient's case. Moreover, I have authorization from our Institutional Ethics Committee and can provide you with it upon request.

## Consent

Written informed consent was obtained to publish this case report and accompanying images. On request, a copy of the written consent form is available for review by the editor‐in‐chief of this journal.

## Conflicts of Interest

The authors declare no conflicts of interest.

## Data Availability

The data underlying this case report will be shared on request with the corresponding author.
